# Supplementary figures and images for: Antagonistic regulation of salt and sugar chemotaxis plasticity by a single chemosensory neuron in Caenorhabditis elegans
Source: PLoS Genet. 2023 Sep 5;19(9):e1010637. doi: 10.1371/journal.pgen.1010637 (PMC10503759; doi:10.1371/journal.pgen.1010637)

A

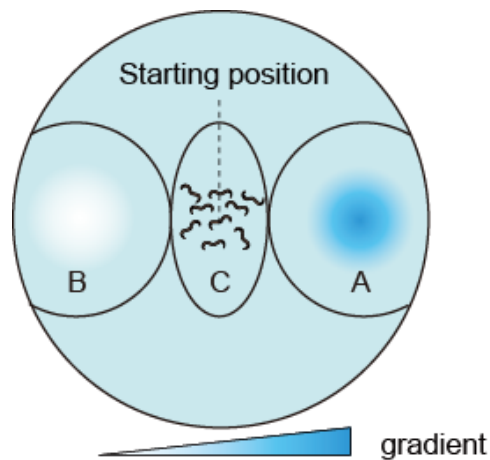

$$\text{Chemotaxis index} = \frac{N_A - N_B}{N_{All} - N_C}$$

B

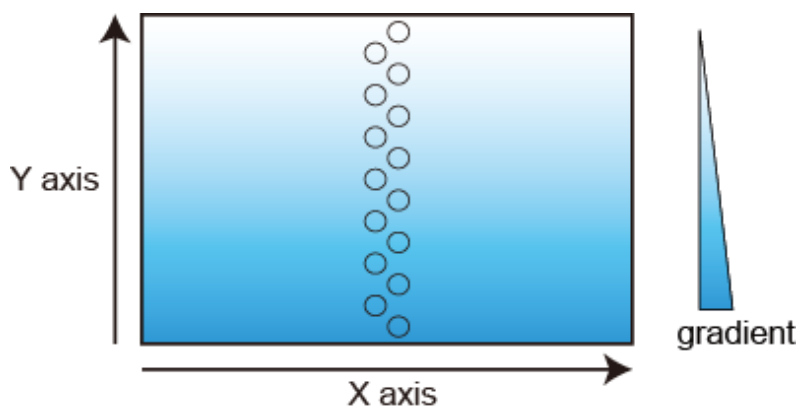

C

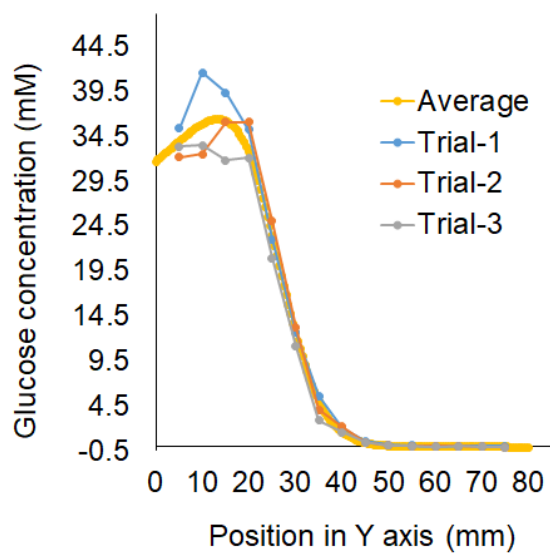

Supplement: S1 Fig — (A) Schematic of a chemotaxis-test plate used for chemotaxis assays, except for tracking analyses of worm locomotion. Fifteen to two hundred worms were used in each assay. (B) Schematic for a locomotion-test plate used for tracking analyses using a multiworm-tracking system. Circles represent areas excised for measurement of glucose concentrations. Thirty to fifty worms were used in each assay. (C) Glucose concentrations relative to positions on the y-axis in a test plate shown in B. Each curve from the three trials and the average curve smoothed by a cubic spline method are shown. (PDF) [file pgen.1010637.s004.pdf]

### Calcium imaging of ASER

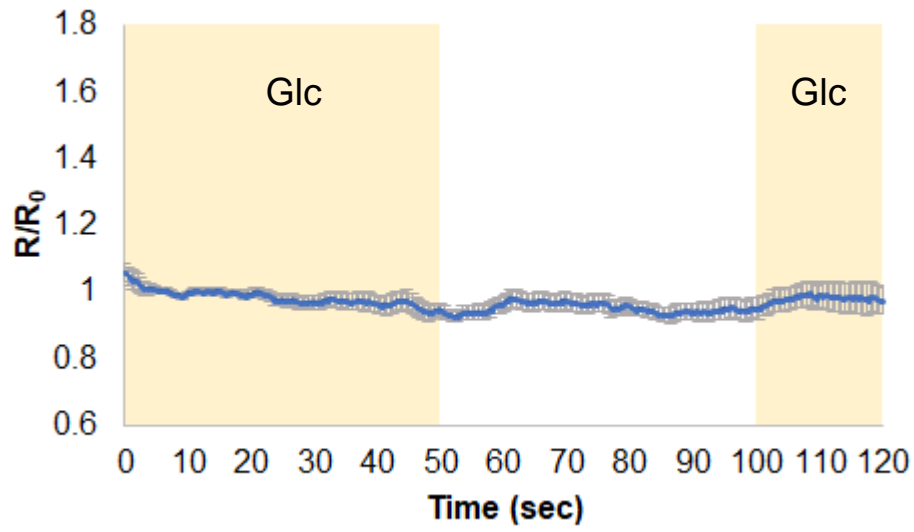

Supplement: S2 Fig — Calcium responses of ASER upon glucose concentration changes after conditioning with glucose in the presence of food. Time course of the average fluorescence intensity ratio (YFP/CFP) of YC2.60 relative to the basal ratio (R/R0) in AESR. The glucose concentration was switched from 15 to 0 mM at 50 s and then returned to 15 mM at 100 s. (PDF) [file pgen.1010637.s005.pdf]

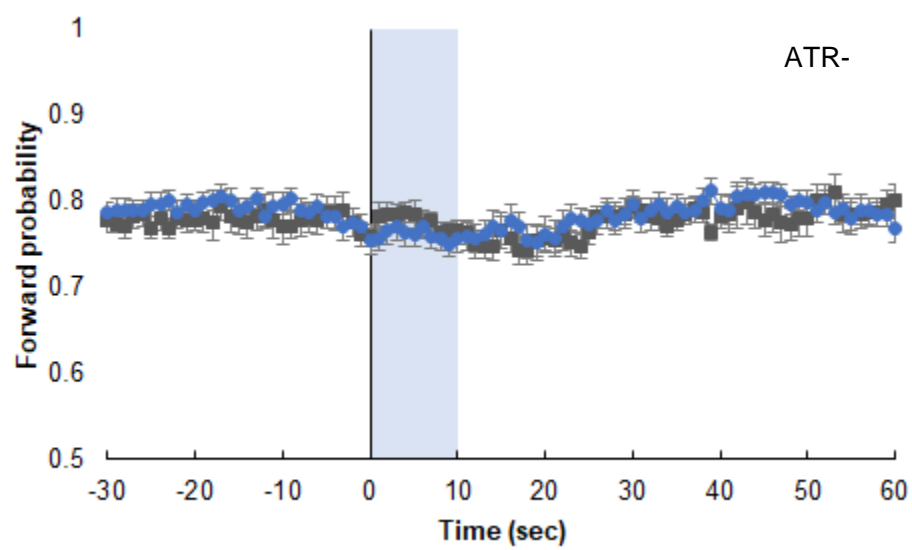

Supplement: S3 Fig — After feeding conditioning with (blue traces) or without (gray traces) glucose, probabilities of forward movement were monitored in worms expressing ChR2 in ASEL on agar plates, containing 5 mM glucose. Blue light was illuminated for 10 s (shaded in blue). As a control experiment in Fig 3A, all-trans retinal (ATR) was not applied during conditioning. (PDF) [file pgen.1010637.s006.pdf]

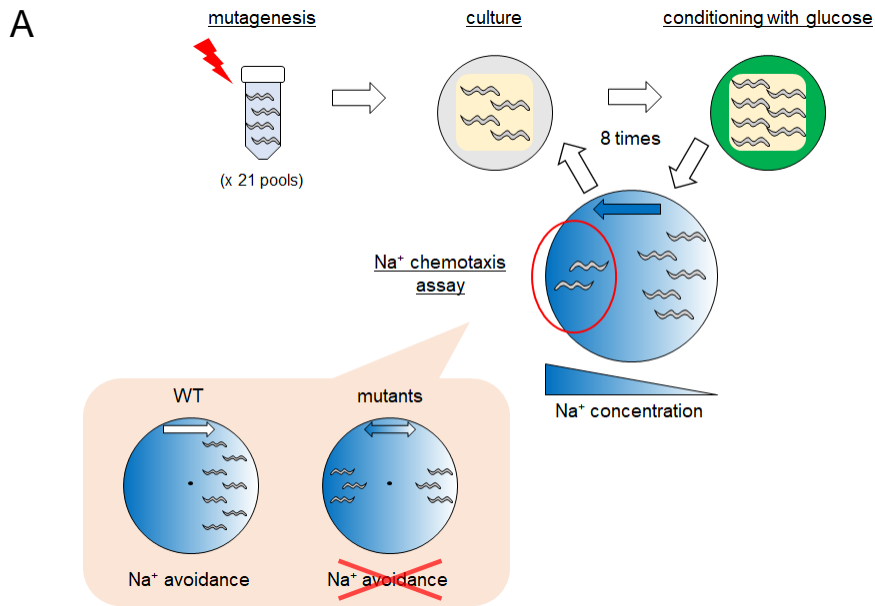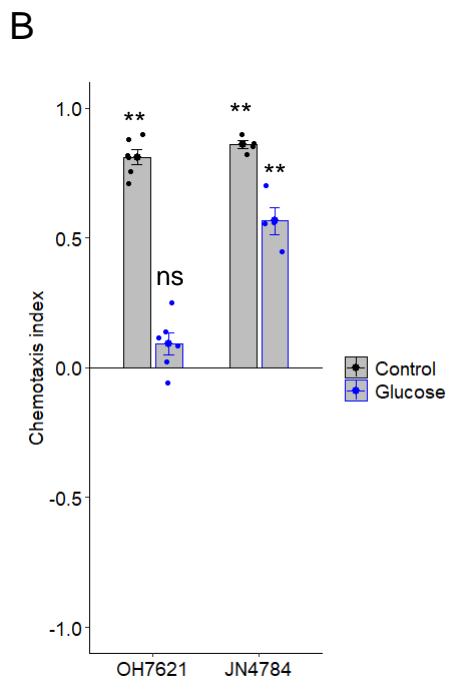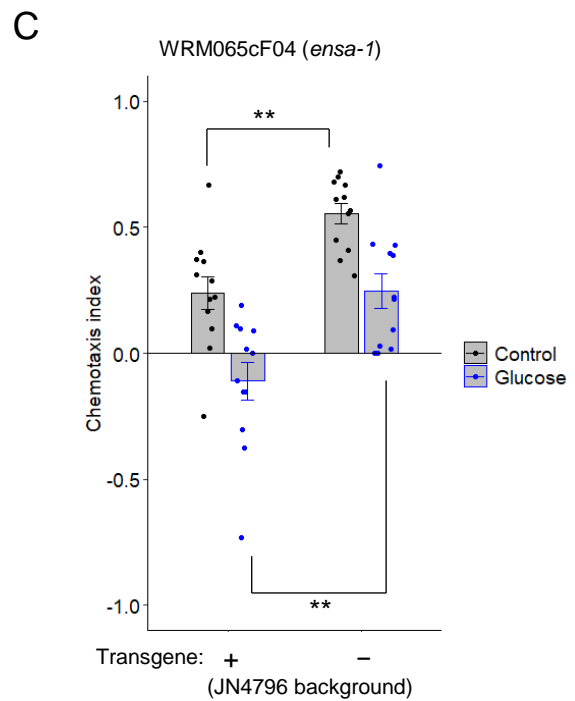

Supplement: S4 Fig — (A) Schematic of screening for mutants defective in Na+ avoidance after glucose conditioning. See detail in the Methods section. (B, C) After feeding conditioning with (“glucose”) or without (“control”) glucose was complete, chemotaxis to Na+ was tested. Original (OH7621) and isolated mutant (JN4784) strains were used (B). The JN4796 strain, which was isolated by outcrossing JN4784 with the wild type N2, with (+) or without (−) the fosmid, WRM065cF04, including ensa-1 gene. See exact genotypes in S1 Table. Bars represent mean values; error bars represent SEM. n = 4–6 (B), 12 (C). One-sample, two-tailed t-test against zero value with Bonferroni correction: **P < 0.01 (B). Two-tailed Welch’s t-test: **P < 0.01 (C). (PDF) [file pgen.1010637.s007.pdf]

A

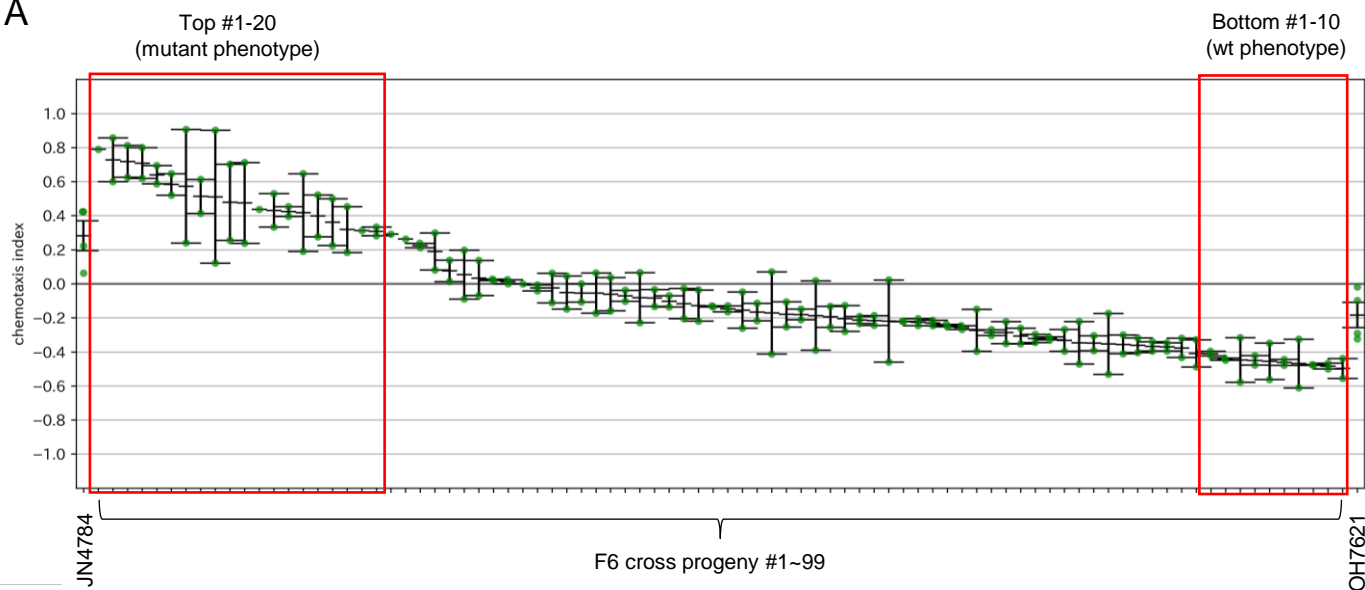

B

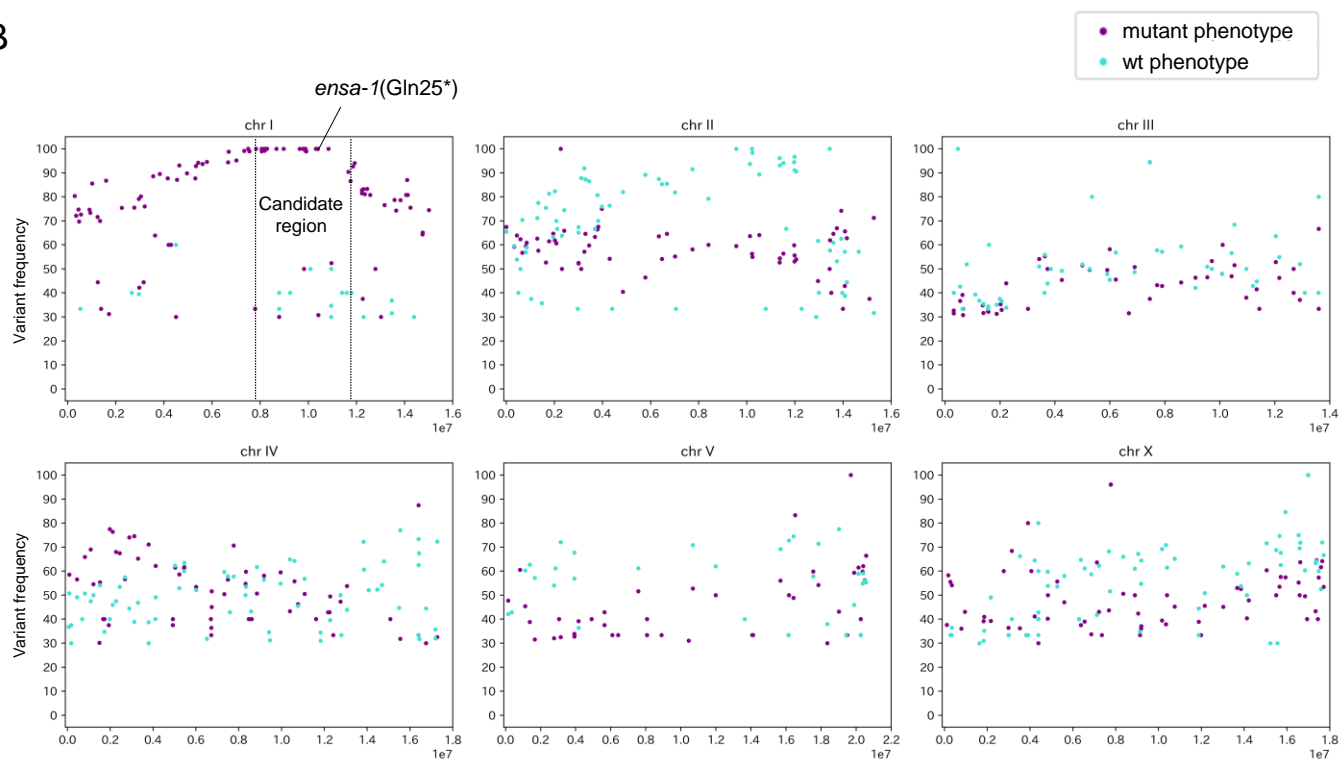

Supplement: S5 Fig — (A) After feeding conditioning with glucose, chemotaxis to Na+ was tested in JN4784, OH7621 and 99 of F6 cross progenies, which were isolated after crossing JN4784 with OH7621. Bars represent mean with SEM. n = 4 (JN4784, OH7621), 2 (cross progenies). (B) Variant frequencies in cross-progeny populations showing mutant (purple) or wild type (sky blue) phenotype. The horizontal axis represents physical positions on each chromosome. (PDF) [file pgen.1010637.s008.pdf]
