# Supplementary material for: Antagonistic regulation of salt and sugar chemotaxis plasticity by a single chemosensory neuron in Caenorhabditis elegans
Source: PLoS Genet. 2023 Sep 5;19(9):e1010637. doi: 10.1371/journal.pgen.1010637 (PMC10503759; doi:10.1371/journal.pgen.1010637)
Supplement: Attachment — Submitted filename: Response letter_Tomioka_et_al_final3.docx [file pgen.1010637.s009.docx]

**Point-by-point responses to the referees’ comments**

We really appreciate all the reviewers for carefully reading our manuscript and pointing out the constructive and important comments. By the revision according to the reviewers’ comments, our manuscript was greatly improved. Our point-by-point responses to the reviewers’ comments were as follows.

**Reviewer #1**

**Major Comments:**

*Does conditioning with any monosaccharide cause attraction? e.g. Fructose conditioning and glucose attraction or vice versa?*

According to the reviewer’s suggestion, we added the results of glucose or fructose chemotaxis assay after fructose or glucose conditioning, respectively. C. elegans showed attraction to these monosaccharides after the conditioning. We also confirmed that C. elegans showed avoidance of Na+ after conditioning with fructose as well as glucose. Therefore, they are attracted to the monosaccharides glucose and fructose and avoid Na+ after conditioning with either monosaccharide. The results are shown in Fig. 1C and Fig. 4B, fourth graph, and line 136-138, page 8 and line 303-306, page 20.

*Section starting Line 146 - The rationale for examining the ASEs is not well justified. Why not examine any other sensory neuron? Did the authors test any other neuron(s)?*

In fact, we first conducted cell-specific rescue experiments using the sensory cilia-defective dyf-11 mutant to explore chemosensory neurons important for glucose chemotaxis. As the rescue of cilium function of ASEL was sufficient for the rescue of glucose chemotaxis, we next examined the cell ablation experiment and calcium imaging of ASEL. We edited the description in the result section (line 162-198, page10-12) and rearrange the panels in Fig 2 in the order we actually examined. We also added the result of glucose chemotaxis using the dyf-11 mutant in which dyf-11 function (cilium function) is rescued in chemosensory neurons other than ASE by the odr-4 promoter (Fig. 2A, forth block). Glucose chemotaxis was significantly rescued in the dyf-11 mutant by dyf-11 expression in chemosensory neurons other than ASEL. Therefore, multiple chemosensory neurons, including ASEL, could regulate glucose chemotaxis. We added this suggestion in the result section (line 171-174, page 11) and added discussion about the possibility that multiple chemosensory neurons may regulate glucose chemotaxis plasticity (line 485-488, page33-34).

*The authors tested the role of synaptic neurotransmission in glucose responses but not neuropeptides using an unc-31 mutant. This needs to be addressed.*

Thank you for the suggestion. Regarding functions of ASEL in glucose chemotaxis, we have shown that rescue of the cilium function only in ASEL among the ciliated sensory neurons was sufficient for the rescue of glucose chemotaxis after glucose conditioning (Fig. 2A). This finding suggests that glucose sensing only by ASEL among the ciliated sensory neurons is sufficient for glucose chemotaxis. The calcium imaging experiment showing ASEL responds to decreases in glucose concentrations was consistent with this notion (Fig. 2C and D). We also confirmed that ASEL is required for glucose attraction by the cell-ablation experiment (Fig. 2B). Therefore, we concluded that ASE responds to glucose and important roles in glucose chemotaxis.

Based on the finding that ASEL can respond to glucose in the unc-13 synaptic transmission deficient mutant at the same level as that in the wild type, we show that synaptic neurotransmission is not required for glucose response in ASEL. However, as the reviewer suggested, the relevance of unc-13-independent communication, such as neuropeptidergic signaling, is unexplored. Therefore, we added the precise explanation of this experimental result as follows. “The ASEL neuron of an unc-13 mutant, in which synaptic transmission is largely disrupted due to exocytosis defects, responded to the decrease in glucose concentration at the same level as that in the wild type (Fig. 2C bottom, D). The result suggests that synaptic transmission is not required for the glucose response in ASEL, although we cannot rule out the possibility that unc-13-independent communication, such as neuropeptidergic signaling, modulates the ASEL response.” (line 192-198, page 12) In addition, we weaken our claim that ASEL senses glucose and stated that ASEL responds to glucose (line 102, line 110, line 485).

Moreover, relevance of neuropeptide signaling in the regulation of glucose chemotaxis is valuable to discuss their regulatory mechanisms in the neural circuits and inter-tissue levels. In mammals, glucose is sensed by peripheral organs, such as the intestine and pancreatic beta cells, and modulates peptidergic signaling to reduce blood glucose levels. There might be similar mechanisms in C. elegans. Glucose may be received by cells other than ciliated sensory neurons, such as those in the nervous system and peripheral tissues, and may modulate glucose chemotaxis through neuropeptides. Therefore, we added discussion of the possible function of neuropeptidergic signaling in the regulation of glucose chemotaxis in line 476-491, page 33-34. We would like to elucidate the relevance of neuropeptidergic signaling in the regulation of glucose chemotaxis as a topic for future research.

*More context in the discussion regarding the importance of such mechanisms in the worms’ natural environment*

We added discussion regarding why worms show innate avoidance of monosaccharides as follows. “Although these monosaccharides are important for energy production, high concentrations of glucose are known to be harmful to animals, including *C. elegans*. High-glucose conditions can cause mitochondrial damage, generate oxidative stress and reduce the life span in *C. elegans* [31, 32]. Therefore, the avoidance of high concentrations of glucose would be beneficial for survival. On the other hand, in the natural environment, *C. elegans* is abundant in microbe-rich rotting plant matter, such as deposing fruits and stems [33], where high concentrations of sugar could exist. Monosaccharides may be used as chemical cues to memorize previous feeding locations, and worms are attracted to and remember these locations in the natural environment. *C. elegans* may flexibly alter its behavioral responses to various environmental signals, including hazardous substances, to obtain food in harsh natural environments.” (line 450-460, page32)

**Minor Comments:**

We appreciate the reviewer for carefully pointing out the errors in the English writing.

*Line 84 change ‘whose sensing’ to ‘ the sensing of which’ Line 111 change ‘on agar’ to ‘on an agar’*

We corrected them.

*Line 149-151 this senstence needs to eb re-worked as it is very confusing ‘Left- and right-sided ASE neurons, ASEL and ASER, respectively, receive different ions and respond to increase and decrease, respectively, in concentrations of ions to promote attraction to those ions [19, 22].’*

We edited this sentence as “ASEL and ASER are reported to receive different ions. ASEL responds to increases and ASER responds to decreases in concentrations of ions to promote attraction to those ions [26].”. Because explanation of left and right positions of ASE is described in the Introduction section, we omitted this explanation in the above sentence.

*Line 154 ‘rapidly ceased’ reads weird. Would reduced be better? Line 183 change ‘the wild type’ to ‘wild type’*

We changed “ceased” to “reduced” and “the wild type” to “wild type”.

*Line 201 ‘**By contrast, it decreased in worms after glucose conditioning’ - what do the authors refer to ‘it’ - define what ‘it’ is.*

This sentence was edited according to the suggestion by the reviewer #2 and #3 as follows and define “it” as “a positive effect in forward locomotion probability”. “By contrast, a positive effect in forward locomotion probability was not observed by optogenetic ASEL activation in worms after glucose conditioning. Rather, ASEL activation caused a decreasing tendency in forward locomotion probability after glucose conditioning.” (line 233-236, page 15)

*Line 267 - change ‘the dyf-11 mutant, in which sensory cilium is rescued only in ASEL.’ to ‘the dyf- 11 mutant, in which dyf-11 expression, and likely cilia function, is rescued only in ASEL’*

Thank you for restating it correctly. We corrected it according to the reviewer’s suggestion.

*Line 276 - define ‘they’*

We changed “they” to “the 2-ASEL strain”.

*Line 370 remove the word ‘the’ before monosaccharides*

We removed “the” before monosaccharides.

*The use of the word authentic promoter is not usual. I presume the authors mean the dyf-11 promoter here. I suggest just calling in the dyf-11 promoter to avoid confusion.*

We changed “authentic promoter” to “dyf-11 promoter”.

*Figure 1A - the number of worms used per assay is not clear*

We added the number of worms used per assay in S1 Figure A and B.

**Methods –**

*Two plasmid DNAs, including Cas9 cDNA under the rgef-1 promoter or ensa-1 sgRNA under the U6 promoter - this doesn’t sound correct. The rgef-1 promoter drives expression in neurons and not the germline.*

We actually used the rgef-1 promoter to drive Cas9 expression and obtained two ensa-1 mutants as described in the method section. As the reviewer pointed out, the rgef-1 promoter is known to drive expression in neurons. In addition, it may drive expression also in the germline, although we have not directly tested it.

**Reviewer #2**

**Major comments:**

**Major comments on experiments**

*The results of Fig. 3A and 3B of ATR+ do not seem to match. I agree that light stimulation significantly increases the forward probability in the naive condition. However, the conditioned result does not look so.*

In the original manuscript, the forward probability change shown in Fig. 3B was calculated by subtracting average forward probability during photostimulation (3s to 7s) by that before photostimulation (-20s to 0s). During 20s period before photostimulation, the forward probability seems to gradually decrease by an unknown effect. So, the forward probability change may include the artifact of this gradual decrease before photostimulation. In the revised manuscript, we calculated the forward probability change by subtracting average forward probability during photostimulation (3s to 7s) by 5 sec period just before photostimulation (-5s to -1s) to reduce this artifact. As a result, the forward probability change upon photostimulation after glucose conditioning significantly decreased in the presence of ATR when compared with zero value, whereas no statistical change was observed in the absence of ATR (Fig. 3B). On the other hand, no statistical change was observed when compared between these results of ATR+ and ATR- (Fig. 3B, conditioned). Based on these findings, we weakened our claim and concluded as follows. “By contrast, a positive effect in forward locomotion probability was not observed by optogenetic ASEL activation in worms after glucose conditioning. Rather, ASEL activation caused a decreasing tendency in forward locomotion probability after glucose conditioning.” (line 233-236, page 15)

*I do not agree with the interpretation of the results in Fig. 5. The authors wrote that "PKC-1 function is required for avoidance of Na+ after glucose conditioning." However, (1) a simple interpretation of Fig. 5C and D suggests that the role of pkc-1 is to suppress Na+-taxis in the naive state, and that salt conditioning suppresses the function of PKC-1, allowing Na+-taxis to occur. Furthermore, (2) based on Fig. 5A, B, it can be inferred that the function of pkc-1 is not involved in the response to glucose in the naive state, but rather, it induces attraction to glucose after conditioning.*

Thank you for the suggestion. We edited the interpretation of glucose and Na+ chemotaxis defects after glucose conditioning in the pkc-1(lf) mutant. According to the reviewer’s suggestion, we concluded as follows.

1) Based on the result in Fig. 5B in the revised manuscript, repression of PKC-1 may underlie Na^+^ attraction after NaCl conditioning. (line 349-353, page 23)

2) Based on the result in Fig. 5A in the revised manuscript, PKC-1 is required for glucose attraction after glucose conditioning. (line 345-349, page 23)

3) Based on the result in Fig. 5B in the revised manuscript, PKC-1 is required for Na+ avoidance after glucose conditioning. (line 353-355, page 23)

Furthermore, based on the experiment of PKC-1(gf) expression showing that increased PKC-1 activity in ASEL promoted glucose attraction and Na+ avoidance biases (Fig. 5C, D), we concluded that “These behavioral phenotypes caused by *pkc-1(*gf*)* expression are consistent with the hypothesis that PKC-1 activity in ASEL promotes both glucose attraction and Na^+^ avoidance after high-glucose conditioning. (line 361-363, page 23-24)

*Fig. 6C: If we simply predict the double mutant phenotype, all indices may be around 0.5-0.7, but that was not the case. Of course, experiments do not always go as expected. Still, I would like an explanation of the possible interpretations.*

Thank you for the suggestion. We carefully interpret the phenotypes of the pkc-1; ensa-1 double mutant. First, based on the additive effect of the pkc-1; ensa-1 double mutation on decrease in Na+ attraction after glucose conditioning (Fig. 6C), we concluded that “This additive effect of *pkc-1* and *ensa-1* mutations suggests the redundant function of *pkc-1* and *ensa-1* in decrease in Na^+^ attraction after glucose conditioning*.*” (line 404-406, page 27). As the reviewer pointed out, the double mutant showed unexpected phenotype. Na+ chemotaxis after Na+ conditioning significantly decreased in the double mutant. We added the description of this phenotype and interpreted this phenotype as “PKC-1 and ENSA-1 are required also for Na^+^ attraction after NaCl conditioning.” (line 407-408, page 28).

**Minor comments for experiments**

*line 86 should be read as: "Although naive worms avoid these monosaccharides, they are attracted..."*

We corrected it according to the reviewer’s suggestion.

*line 118: Does "after feeding conditioning without glucose" mean naive?*

Yes. Worms after feeding conditioning without glucose is the same as naïve worms. We changed this sentence as “naive worms showed significant avoidance of glucose” to avoid confusion. (line 128-129, page 7)

*line 167: Original paper(s) for dyf-11 cloning should be cited as well.*

We cited the original paper for dyf-11 cloning. The paper is as follows.

24. Kunitomo H, Iino Y. Caenorhabditis elegans DYF-11, an orthologue of mammalian Traf3ip1/MIP-T3, is required for sensory cilia formation. Genes Cells. 2008;13(1):13-25. doi: 10.1111/j.1365-2443.2007.01147.x.

*line 212: "after increasing and decreasing glucose concentration" was not described in the Figure.*

Thank you for the suggestion. We actually observed frequencies of pirouette according to the glucose concentration changes on agar plates with glucose concentration gradients (Fig. 3C) and quantified the pirouette frequency during decreasing glucose concentration (dC/dT < 0) relative to that during increasing glucose concentration (dC/dT > 0) as “Pirouette index” (Fig. 3H). Therefore, “Pirouette frequency increased and decreased after increasing and decreasing glucose concentration” in the original manuscript is incorrect. We corrected as “Pirouette frequency increased during increasing glucose concentration (dC/dT > 0) compared to decreasing glucose concentration (dC/dT < 0) in wild type worms without conditioning (Fig. 3C and H first block, naïve).”. (line 247-249, page 16)

*Line 339: How about changing Fig. 6A and B?*

According to the reviewer’s suggestion, we changed Fig. 6A and B in the revised manuscript.

*line 360: Which promoters were used in Fig. 6D?*

We added the promoter names in the Fig. 6D.

*line 431: Why was the food bacteria changed? Wouldn't this result in a loss of consistency between behavior and calcium imaging?*

Thank you for the suggestion. We added the description regarding bacteria differently used for behavior and calcium imaging experiments as follows. “When *C. elegans* is grown with *E. coli* NA22 as a food source, the intestine has a tendency to enlarge, pushing the intestine forward and bringing it closer to the neuronal population. In this case, autofluorescence of the intestine can affect the fluorescence imaging. To avoid this, we used *E. coli* OP50 for calcium imaging. We used the NA22 strain for the behavioral experiments because NA22 has a higher growth rate than OP50, and more worms can be obtained by feeding with the NA22 food source.” (line 537-543, page 36-37)

*line 444: Add "in total".*

Thank you for the suggestion. We added it.

*line 447: I am unfamiliar with CRISPR/Cas system, but I couldn't understand why cas9 cDNA needs to be expressed under rgef-1 promoter for neurons.*

We actually used the rgef-1 promoter to drive Cas9 expression and obtained two ensa-1 mutants as described in the method section. As the reviewer pointed out, the rgef-1 promoter is known to drive expression in neurons. In addition, it may drive expression also in the germline, although we have not directly tested it.

*line 508: Please describe the company name and cat. no. for the ring LED.*

We described the company name (CCS Inc.) and cat. no. (LDR2-90BL) for the ring LED.

*Fig. 2A: Adding the glucose concentration info will be helpful.*

We added the glucose concentration in Fig. 2C (Fig. 2A is changed to Fig. 2C in the revise manuscript).

*Fig. 2C: What does "authentic" mean?*

We changed the “authentic” promoter to the “dyf-11” promoter.

**Writing:**

*The first paragraph of the Introduction seems to be about the general sensory reception of animals, but all the references cited are for C. elegans. This is strange. The references should either include more sources other than C. elegans, or make it clear that the focus is solely on C. elegans from the beginning.*

As the reviewer pointed out, we clearly stated that the focus of this study is on C. elegans in the first paragraph of the Introduction. And then, we carefully explained about two excellent examples of the studies about multiple sensory integration in C. elegans as introduction of this study. (line 63-73, page 4). Further, we also mentioned the relationship between those previous examples and the achievement of our article in Discussion (line 488-491, page 34).

*The second paragraph of the Introduction describes C. elegans' sensory reception, particularly regarding NaCl. However, only papers from the authors' group were cited, despite excellent results on this topic from many other research labs.*

We added the description about associative learning between food status and sensory cues other than NaCl, which are reported by other labs as follows. “Similarly, *C. elegans* memorizes the presence of odors and temperatures and increases attractive responses toward the sensory cues experienced during feeding [12-15]. It is proposed that *C. elegans* memorizes multiple sensory cues during feeding and approaches the environment where it previously fed to increase the probability of obtaining food.” (line 89-92, page 5)

*The last paragraph of the Introduction is problematic. I recommend authors to read "Science Research Writing For Non-Native Speakers Of English" by Hilary Glasman-Deal.*

Thank you for your introduction of the book about science research writing. The sentence structure of the last paragraph of the Introduction was changed. Introduction about taste reception in mammals and C. elegans was moved to the separate paragraph and then the summary of this paper was described in the next (last) paragraph. Please see line 93-117, page 6-7.

The way the figure legends are placed is also inadequate. Generally, the main figure legends should come after the main text. If they are inserted in the main text, they should be placed together with the figures. Additionally, the Sup. Fig. legend should be on the same page as the Sup. Fig.

I am sorry for the difficulty in reading the original manuscript. In the revised manuscript, the main figures were inserted along with the main figure legends in the main text. Sup. Fig. legend was added on the same page as the Sup. Fig.

**Reviewer #3:**

**Main concerns are:**

*Statistical methodology may be incorrect in a number of instances.*

We corrected statistical methods according to the reviewer’s suggestions. Please see the details below.

*The Discussion lacks a clear summary of the working model this paper substantiates.*

We added a summary figure of the regulation of glucose and Na+ chemotaxis responses by the ASEL neuron in Fig. 7. A clear summary of the working model is described in the caption of Fig. 7. This figure was referred in each paragraph in the Discussion section.

*The paper is hard to read as it is quite terse, with intervening steps of experimental logic and interpretation left for the reader to discover.*

We edited description in the Result section as follows to improve the readability of the paper.

1) We carefully explained the experimental logic and interpretation regarding discovery of the important role of ASEL in glucose chemotaxis regulation. (line 162-198, page 10-12).
2) We carefully explained the experimental logic and interpretation regarding locomotion analyses after optogenetic stimulation of ASEL (line 225-241, page 15).
3) We carefully explained the phenotypes caused by the pkc-1 mutation and overexpression of PKC-1(gf) in glucose and Na+ chemotaxis plasticity and stated the clear interpretations based on the mutant phenotypes. (line 345-365, page 23-24)
4) We carefully explained the behavioral phenotype in the double mutant of pkc-1 and ensa-1 and stated the interpretations based on those genetic interactions. (line 402-408, page 27-28)

**MAJOR**

*Figs. 2C, 4A, 4B, 4C, 5A, 5B, 6D, etc. The authors frequently use statistical comparisons between treatment groups to demonstrate the presence or absence of attraction or repulsion. These seem incorrect. The appropriate test would be difference from zero (see Fig. 1A). This is a non-trivial matter as some of the author's claims may turn out to be unsupported by the data when the appropriate statistics are used. For example, the claim of Glc attraction in unconditioned egl-30 may not hold up (Fig. 5A). The same concern applies to the claim of Glc attraction in unconditioned animals expressing PKC-1(gf) in ASEL (Fig. 5B). The authors should check all figures for other instances of this error.*

Thank you for pointing out the important point. According to the reviewer’s suggestion, statistical comparisons between zero were carried out in panels B and C in Fig. 1, panels A and B in Fig. 2, panels B and H in Fig. 3, all panels in Fig. 4, panels A, B in Fig. 5, panels A-D in Fig. 6 in the revised manuscript to show whether worms show attraction or avoidance. The result of glucose chemotaxis in the egl-30 mutant (Fig. 5A) was omitted because we do not focus on an upstream regulator of PKC-1 in this paper.

*Fig. 3A conditioned. The claim of reduced forward probability in the conditioned group upon ChR2 activation of ASEL looks like it might be an artifact of the analysis. The authors computed the difference between mean P-forward in the entire prestimulus baseline period and mean P-forward during the stimulus. This difference is amplified by the fact that the prestimulus baseline is elevated for some reason. If, instead, the authors took as the prestimulus baseline the 5 sec period before the stimulus, the effect would likely disappear.*

Thank you for the constructive comment. We calculated the forward probability change by subtracting average forward probability during photostimulation (3s to 7s) by 5 sec period just before photostimulation (-5s to -1s) to reduce the artifact that the reviewer pointed out. As a result, the forward probability change upon photostimulation after glucose conditioning significantly decreased in the presence of ATR when compared with zero value, whereas no statistical change was observed in the absence of ATR (Fig. 3B). On the other hand, no statistical change was observed when results of ATR+ and ATR- were compared (Fig. 3B, conditioned). Based on these findings, we weakened our claim and concluded as follows. “By contrast, a positive effect in forward locomotion probability was not observed by optogenetic ASEL activation in worms after glucose conditioning. Rather, ASEL activation caused a decreasing tendency in forward locomotion probability after glucose conditioning.” (line 233-236, page 15)

*Fig. 3A Naive. Could the authors please discuss the apparent rebound effect after the stimulus. Maybe there's a rebound effect in the Naive group as well. Perhaps these issues could be clarified by showing the full 40 sec of pre- and poststimulus data.*

To clarify the rebound effect after switching off the blue light, we showed full traces with 80 sec interval between photostimulation (ATR+: Fig. 3A and shown below, ATR-: Supplemental Fig. S3). In the naïve worms (gray trace), the forward locomotion probability tended to decrease after switching off the blue light and return to the baseline within 30 s. We suspect that the decreased forward probability change may result from hyperpolarization effect as a rebound reaction. On the other hand, no rebound effect seems to be observed in the glucose-conditioned worms (blue trace). We carefully explained the results of the ASEL photostimulation and added the possible explanation regarding the rebound effect (line 227-233, page 15).

*Discussion. This section might be improved by a summary figure showing how the effects of conditioning on ASEL interact with the PKC-1 and ENSA-1 pathways to regulate attraction and repulsion. See also comment below (regarding lines 305-310).*

According to the reviewer’s suggestion, we added a summary figure showing how ASEL responses and PKC-1 and ENSA-1 in ASEL regulate attraction and avoidance of glucose and Na+ in Fig. 7.

**MINOR**

*56-67. The theme of sensory motor integration, mentioned in the introduction, is under developed in the main text.*

We edited the description regarding integration of multiple information in the first paragraph in the introduction section to improve the readability. In this paragraph, we carefully explained two examples of multiple sensory integration. (line 63-73, page 4) We also mentioned the relationship between these examples and the achievement of our article in Discussion (line 488-491, page 34).

*Could the authors please explain why they find repulsion rather than attraction to glucose, as previously reported in reference [20]*

We carefully read this reference, but it is not described how worms used for glucose chemotaxis assay were cultivated. If the worms were cultivated on the media that includes high glucose, they were attracted to glucose. But we do not know whether glucose contained in the cultivation media in this previous experiment. So, it was difficult to precisely compare this report with our study.

*189 and captions. "Authentic" promoter is confusing. Maybe "native" promoter would be more clear.*

Thank you for the suggestion. We changed “authentic” promoter to “dyf-11” promoter to avoid confusion.

*212. "Pirouette frequency increased and decreased after increasing and decreasing glucose concentration" is a bit confusing. Strictly speaking, Fig. 3C shows that pirouette frequency was above baseline (at dC/dt = 0) for dC/dt > 0 and below baseline for dC/dt < 0.*

We actually observed frequencies of pirouette according to the glucose concentration changes on agar plates with glucose concentration gradients (Fig. 3C) and quantified the pirouette frequency during decreasing glucose concentration (dC/dT < 0) relative to that during increasing glucose concentration (dC/dT > 0) as “Pirouette index” (Fig. 3H). Therefore, “Pirouette frequency increased and decreased after increasing and decreasing glucose concentration” in the original manuscript is incorrect. We corrected as “Pirouette frequency increased during increasing glucose concentration (dC/dT > 0) compared to decreasing glucose concentration (dC/dT < 0) in wild type worms without conditioning (Fig. 3C and H first block, naïve).”. (line 247-249, page 16)

*240. "dC/dt rank" is confusing, because nothing was ranked. Rather, there are two DOMAINS, dC/dt > 0 and dC/dt < 0, and the pirouette index is the difference in mean pirouette frequency in these two domains.*

We changed “dC/dt rank” to “dC/dt domain” and the description to “Migration bias was quantified by using the pirouette index, defined as the difference of mean probability of pirouette between negative dC/dT domain and positive dC/dT domain.” (line 275-277, page 18)

*272. Should this read "(Fig. 4C, THIRD block)"?*

The corresponding description is as follows. “By contrast, recovery of the sensory cilium of ASEL was sufficient for attraction and avoidance of Na+ after NaCl and glucose conditioning, respectively, suggesting that sensory perception by ASEL is required for two directions of Na+ chemotaxis after NaCl and glucose conditioning (Fig. 4C, the fourth block).” (line 310-314, page 20) This sentence is described about the dyf-11 strain expressing dyf-11 in ASEL by the gcy-7 promoter. Therefore, the reference data is the fourth block in Fig. 4C. To avoid confusion, explanation of the promoter and the dyf-11-epxressing neuron type was added below in the Fig. 4C graph.

*273-4. Indicate which figure panel contains the data supporting this conclusion.*

The data which supports this conclusion is Fig. 4C. We edited this sentence to carefully explain and added the reference panel as follows. “Unlike wild type worms and the *dyf-11* mutant whose sensory cilium was recovered by the *dyf-11* promoter, the *dyf-11* mutant whose sensory cilium of ASEL was recovered showed strong Na^+^ attraction even without NaCl conditioning (Fig. 4C)” (line 314-316, page 20)

*Fig. 4C. Please explain why the authentic and ASEL-selective promoters give different results in dyf-11 rescue.*

*277. Please explain how the results with 2-ASEL strain imply something about ASER function.*

Based on the finding that the ASEL-selective dyf-11 rescue line, but not the dyf-11 rescue line by the dyf-11 (authentic) promoter, showed strong Na+ attraction even without NaCl conditioning, we speculate that sensory perception from ciliated sensory neurons other than ASEL represses Na^+^ attraction in naive wild type worms. In the 2-ASEL strain, ASER is converted to ASEL identity, thus this strain does not have functional ASER. Because the 2-ASEL strain showed Na+ attraction even without NaCl conditioning similar to the ASEL-selective dyf-11 rescue line, we speculate that ASER may function to reduce Na^+^ attraction in conditions without high NaCl exposure in the presence of food. These explanations were described in the revised manuscript as follows.

“Unlike wild type worms and the *dyf-11* mutant whose sensory cilium was recovered by the *dyf-11* promoter, the *dyf-11* mutant whose sensory cilium of ASEL was recovered showed strong Na^+^ attraction even without NaCl conditioning (Fig. 4C), which implies that sensory perception from ciliated sensory neurons other than ASEL represses Na^+^ attraction in naive wild type worms. We noticed that a similar phenotype was observed in worms whose neuronal identity of ASER is converted to ASEL identity (2-ASEL strain) [26, 29]. The ASEL-strain showed strong attraction to Na^+^ with or without NaCl conditioning, and glucose conditioning caused Na^+^ avoidance (Fig. 4D). These data imply that ASER may function to reduce Na^+^ attraction in conditions without high NaCl exposure in the presence of food.” (line 314-322, page 20-21)

*305-310. Can the authors please provide a conceptual model of how increased/decreased synaptic output from ASEL or ASER explains the changes in chemotaxis?*

Thank you for the important question. In this study, we showed that changes in synaptic output from ASEL may underlie glucose and Na+ chemotaxis plasticity after high-glucose conditioning by the optogenetic ASEL stimulation experiment. So far, it is unclear how altered synaptic output from ASEL may change chemotaxis of glucose and Na+ after high-glucose conditioning. This is one of important questions we should elucidate in future study.

*339. "Increased Na attraction" relative to what other treatment group?*

We changed this sentence as follows. “Two insertion/deletion mutants of *ensa-1*, *pe4791* and *pe4792*, which were obtained by using a CRISPR/Cas9-based method, showed significant Na^+^ attraction in worms without conditioning.” (line 394-397, page 27) Two ensa-1 mutants, pe4791 and pe4792, showed significant Na+ attraction compared to zero value in worms without conditioning, whereas naïve wild-type animals did not show no significant Na+ attraction. We clearly stated this phenotype.

*351. The authors note that PKC-1 is localized to the presynaptic region in ASER. Where is PKC-1 expressed in ASEL?*

We have not observed subcellular localization of PKC-1 in ASEL. Detailed cell biological functions of PKC-1 and ENSA-1 in ASEL is an important question we should elucidate in future study.

*352. The axon in Fig. 6E is not visible.*

The below picture in 6E was replaced to the one the axon is more visible (Fig. 6E).

*371. What might be the source of monosaccharides in the natural environment? Would bacteria be associated with them?*

We added discussion regarding the source of monosaccharides in the natural environment and the possibility that bacteria would be associated with them as follows. “On the other hand, in the natural environment, *C. elegans* is abundant in microbe-rich rotting plant matter, such as deposing fruits and stems [33], where high concentrations of sugar could exist. Monosaccharides may be used as chemical cues to memorize previous feeding locations, and worms are attracted to and remember these locations in the natural environment. *C. elegans* may flexibly alter its behavioral responses to various environmental signals, including hazardous substances, to obtain food in harsh natural environments.” (line 454-460, page 32)

*400. in _an_ opposite direction.*

We corrected this error.

*Fig. 1. Concentration of Na and sugars during conditioning is unclear.*

We added the concentrations of NaCl and sugars during conditioning in the caption of Fig. 1. “(A) After feeding conditioning with (“conditioned”) or without (“naïve”) 100 mM of chemical indicated on the *x*-axis, chemotaxis to the chemical used for conditioning was tested.” (line 144-146, page 9-10)

*Fig. 2A top. The authors might consider using an ANOVA with repeated measures to test for the effect of conditioning. This avoids the potential bias introduced by arbitrarily defining peak and non-peak regions in the traces.*

Thank you for the suggestion. In this study, we wanted to know the peak values soon after glucose concentration changes to quantify the quick response upon concentration change. So, we obtained the peak values within 10 sec after glucose concentration change (during 50-60 sec in Fig. 2C) in each imaging experiment and these data were summarized as box-and-whisker diagrams and dot plots in Fig. 2D left. The statistical analysis using two-tailed Welch’s t-test clearly showed that there was no statistical difference in peak values soon after the stimulus between naïve and conditioned wild-type worms. Because total calcium influx in conditioned worms seemed to be higher than that in naïve worms, we calculated changes in the average calcium levels between before (0-50 sec) and after (50-100 sec) decrease in glucose concentrations and compared them between naïve and conditioned worms (Fig. 2D right). The change in average calcium levels during decreased glucose concentration in conditioned worms was statistically higher than that in naïve worms. Based on these findings, we concluded as follows. “After glucose conditioning, ASEL was activated at the same level as that without conditioning when glucose concentration decreased (Fig. 2C top, D left). By contrast, at low glucose concentration, the calcium level was significantly higher after glucose conditioning than that without conditioning (Fig. 2C top, D right), implying differences in ASEL properties between worms with and without glucose conditioning.” (line 188-192, page 12)

*Fig. 2C Add the label "Promoter" to the line below the x-axis containing "authentic," "ASEL," "ASER."*

We added the promoter names below the graph in the indicated panel (Fig. 2A in the revised manuscript).

*Fig. 3C-G. How was dC/dt computed?*

We first determined the glucose concentration on the agar plate as described in line 599-604, page 39-40. Based on the glucose concentration on the test plate, concentration changes per second (dC/dt) during locomotion were determined. (line 604-606, page 40)

*Fig. 3H. Y-axis is unlabeled. It should be pirouette index? Fig. 4C Add "Promoter" to x-axis label.*

We added the Y-axis label, “Pirouette index” in Fig. 3H and the promoter names to the x-axis label in Fig. 4C.

*Fig. 5B, first block. These data appear to be the same as Fig. 2C. This should be stated; same for any other re-use of data.*

Thank you for the suggestion. As the reviewer pointed out, data of the wild type in Fig. 2A and Fig. 5C in the revised manuscript was the same, because these experiments were simultaneously conducted and the experimental data were split to the two panels. This was stated in the figure caption in Fig. 5C. (line 374-376, page 26) We confirmed that there was no re-use of data in any other figure.

*Fig. 5B, key is cutoff.*

Fig. 5B was moved to Fig. 5C in the revised manuscript. We confirmed Fig. 5C is normally visible in our computer.

*Fig. 1A,B; 5C,D, control groups. There seems to be no chemotaxis to Na ions, which are normally attractive.*

As the reviewer pointed out, wild type worms show no chemotaxis to Na ions after conditioning on NaCl-free NGM plate, which was reported by Wang et al., (2017) (Reference no. 11 in this paper). We have stated how conditioning was conducted in “Methods” as follows. “For conditioning, adult worms were transferred to modified NGM plates, including 100 mM d-glucose, d-fructose, sucrose, or NaCl instead of ~51 mM NaCl in standard NGM, unless otherwise noted, for 4–5 h at 20°C. NaCl-free NGM plates were used for negative control (shown as “naïve” or “control”).” (line 579-582, page 38-39)

*Fig. 6A,B. Might be good to reverse the order of these panels to match the text order.*

Thank you for the suggestion. We reversed the order of Fig. 6A and B.

*Fig. 6C. Could the authors please explain in what respect the double mutant has a stronger phenotype than the single mutants? This is needed to support their claim of parallel/redundant pathways.*

We carefully interpret the phenotypes of the pkc-1; ensa-1 double mutant as follows. “ Both *ensa-1* and *pkc-1* mutants showed significant attraction toward Na^+^ without conditioning (Fig. 6A and C, black bars) and glucose conditioning significantly decreased Na^+^ chemotaxis of these mutants (Figs. 6B and C, compare black and blue bars). By contrast, in the *pkc-1; ensa-1* double mutant, glucose conditioning had no effect on Na^+^ chemotaxis (Fig. 6C second block, compare black and blue bars). This additive effect of *pkc-1* and *ensa-1* mutations suggests the redundant function of *pkc-1* and *ensa-1* in decrease in Na^+^ attraction after glucose conditioning*.* (line 399-406, page 27)

*The authors make the case that worms can use sensation of monosaccharides as a cue signaling the presence of food. Can worms eat/digest environmental sugars?*

We do not know whether worms eat/digest environmental sugars. But biochemical and fluorescent imaging experiments reveal that ambient glucose is incorporated into the body of *C. elegans* (Ref. 32; Ref. 36), although it is unknown how worms incorporate environmental sugars. Based on this evidence, we added discussion regarding the possibility that incorporated glucose may be sensed by peripheral organs and be related to glucose chemotaxis plasticity through hormonal signaling, such as neuropeptide signaling, as follows. “Biochemical and fluorescent imaging experiments have revealed that ambient glucose is incorporated into the body of *C. elegans* [32, 36]. Thus, ambient glucose may be received in peripheral organs also in *C. elegans.* In this paper, we have shown that the ASEL neuron senses glucose and plays important roles in glucose chemotaxis. However, we cannot rule out the possibility that other chemosensory neurons and/or peripheral organs also sense glucose and are involved in the regulation of glucose conditioning-induced chemotaxis. As seen in the examples of behavioral control based on multiple information processing introduced at the beginning of the paper [4, 6], neuropeptidergic signaling in the nervous system or inter-tissue interaction may be involved in feeding experience-dependent glucose chemotaxis.” (line 482-491, page 33-34)
