# Supplementary material for: Antagonistic regulation of salt and sugar chemotaxis plasticity by a single chemosensory neuron in Caenorhabditis elegans
Source: PLoS Genet. 2023 Sep 5;19(9):e1010637. doi: 10.1371/journal.pgen.1010637 (PMC10503759; doi:10.1371/journal.pgen.1010637)
Supplement: Attachment — Submitted filename: Response letter-fin-2.docx [file pgen.1010637.s010.docx]

**Point-by-point responses to the reviewers’ comments**

We really appreciate the reviewers’ constructive comments and suggestions. We addressed the concerns of the reviewers. We believe that the paper is improved and is now suitable for publication.

**--- Reviewer 1**

*The authors have satisfactorily answered my initial concerns except for the justification for examining the role of the ASEs. In response to my original question the authors state "In fact, we first conducted cell-specific rescue experiments using the sensory cilia-defective dyf-11 mutant to explore chemosensory neurons important for glucose chemotaxis." and "We also added the result of glucose chemotaxis using the dyf-11 mutant in which dyf-11 function (cilium function) is rescued in chemosensory neurons other than ASE by the odr-4 promoter"*

*However, it is not at all clear why the authors examined ASE function. Why not any other chemosensory neuron? As the authors performed cell-specific rescue experiments for other neurons the why not present these data? The jump from using the dyf-11 and odr-4 promoters and then looking at the ASEs is unclear. This needs to be explained better and/or the cell-specific data shown.*

We apologize for the unclear description regarding the reason why we intended to examine the ASE function in glucose chemotaxis learning. Unfortunately, we have not examined the requirement of dyf-11 in particular classes of chemosensory neurons except for ASE in the regulation of glucose chemotaxis learning. Therefore, instead we would like to better explain the reason for focusing on ASEs in the regulation of glucose chemotaxis learning. As shown in the Introduction, we have been focusing on the functions of ASE mainly in chemotaxis toward NaCl. When we found that C. elegans showed behavioral plasticity to glucose, we were simply interested in whether glucose is also received in ASE. In addition, we were interested in examining a possible interaction between multiple chemical cues received in ASE, because ASE senses various water-soluble chemicals, such as salts, nucleotides and vitamins. As described in the response letter in the first revision, we first tested whether dyf-11 (sensory function) is required in ASE or chemosensory neurons other than ASE for glucose chemotaxis and found that the rescue of dyf-11 (sensory function) in ASEL is sufficient for glucose chemotaxis. Therefore, we next examined the ASEL response to glucose using calcium imaging and interaction between Na+ and glucose, which are both responded by ASEL, using behavioral analyses. To clarify the reason for focusing on the ASE neurons, we added the following description before the explanation of the dyf-11 rescue experiments (line 173-180, page 11).

“*C. elegans* has 11 types of chemosensory neurons, the ciliated endings of which are exposed to the outside and can directly sense external chemicals in the amphid, inner labial and phasmid sensory organs [17]. Among those chemosensory neurons, we focused on the ASE classes of chemosensory neuron in glucose chemotaxis, because ASE neurons play major roles in chemotaxis to various water-soluble chemicals, such as NaCl, cAMP and biotin [18, 25], and we intended to examine a possible interaction between multiple chemical cues received in a particular class of chemosensory neurons.”

We also emphasize that we do not exclude the possibility that chemosensory neurons other than ASE play roles in glucose chemotaxis. Indeed, we have stated as follows.

“multiple chemosensory neurons, including ASEL, could regulate glucose chemotaxis.” (line 184-185, page 11).

“the ASEL neuron is required for glucose attraction after glucose conditioning. On the other hand, glucose avoidance seems to be mainly regulated by sensory neurons other than ASEL” (line 188-190, page 11-12)

“In this paper, we have shown that the ASEL neuron responds to glucose and plays important roles in glucose chemotaxis. However, we cannot rule out the possibility that other chemosensory neurons and/or peripheral organs sense glucose and are involved in the regulation of glucose conditioning-induced chemotaxis.” (line 515-518, page 35)

**--- Reviewer 2**

*The authors have adequately addressed most of my previous comments. Unfortunately, however, I still have a few comments:*

*Relatively major:*

1. *Line 404: I do not agree that the effects of pkc-1 and ensa-1 are additive. The double seems statistically similar to pkc-1.*

We modified the description regarding Na+ chemotaxis phenotypes of the pkc-1; ensa-1 double mutant (line 418-435, page 28-29). As the reviewer suggested, the pkc-1 single mutant and the pkc-1; ensa-1 double mutant showed similar phenotypes: Both mutants showed significant attraction to Na+ in the naïve state and after glucose conditioning. However, glucose conditioning significantly reduced Na+ chemotaxis in the pkc-1 mutant, whereas glucose conditioning had no significant effect on the pkc-1; ensa-1 double mutant. In addition, the double mutant showed decreased Na+ chemotaxis in the naïve condition compared to the single mutants of pkc-1 and ensa-1. Based on these different phenotypes of the pkc-1; ensa-1 from those of the single mutants, we concluded that “ENSA-1 functions in parallel with PKC-1 in the regulation of Na+ chemotaxis under naïve conditions and Na+ chemotaxis plasticity after glucose conditioning”.

2. *Line 407: I suppose that pkc-1 and ensa-1 play redundant roles, and that a lack of both causes the apparent phenotype.*

We modified the description of the Na+ chemotaxis phenotype of the pkc-1; ensa-1 mutant after NaCl conditioning (line 420-424, page 28). As the reviewer suggested, we clearly stated that pkc-1 and ensa-1 plays redundant roles in Na+ attraction as follows. “redundant functions of PKC-1 and ENSA-1 are required for Na^+^ attraction after NaCl conditioning”.”

3. *Figure 7: According to Figure 5, pkc-1 and ensa-1 also seem to play roles in the naive condition, which should be properly shown in the drawing.*

As the reviewer pointed out, pkc-1 and ensa-1 mutants showed significant attraction toward Na+ in the naïve condition unlike the wild type (Fig. 5B and Fig. 6A). Although we clearly showed that ensa-1 functions in ASEL in Na+ avoidance after glucose conditioning by cell-specific rescue experiments, ensa-1 expression in ASEL did not rescue this Na+ attraction phenotype in the naïve condition in the ensa-1 mutant (Fig. 6D, right). Because the functional site of ensa-1 in the naïve state is obscure, it was difficult to be shown in the drawing in Fig. 7, which shows the ASEL function in glucose and Na+ chemotaxis. The functions of ENSA-1 and PKC-1 in the naïve state is needed to further explore in future study. In this study, instead of showing the PKC-1 and ENSA-1 functions in naïve condition in the drawing, we added description in the caption of Figure 7 as follows. “It should be noted that ENSA-1 and PKC-1 are required also for repression of Na^+^ attraction in naive conditions and promotion of Na^+^ attraction after NaCl conditioning, although their functional sites are obscure.” (line 472-475, page 33)

Minor:

1. *Line 61: Please consider using "external" instead of "sensory".*

According to the reviewer’s suggestion, “sensory” was replaced with “external”.

2. *The 2nd and 3rd paragraphs in the Introduction: I found it difficult to understand how these paragraphs are related to the results of this manuscript. Adding topic sentences might help clarify this connection.*

To improve the connection between descriptions in the Introduction and the Results, we added some sentences in the 2nd and 3rd paragraphs (line 79-80, 82, 95-98).

3. *Line 113: Could you explain why you consider the information is processed separately?*

In this study, we showed that glucose conditioning and NaCl conditioning have opposite effects on chemotaxis to glucose and NaCl, and normal functions of PKC-1 and ENSA-1 are required for these processes. However, it is still unclear whether conditioning with glucose and NaCl are separately processed to modulate glucose and NaCl chemotaxis in opposite directions. Therefore, we edited this sentence as follows to avoid misleading. “exposures to glucose and Na^+^ during conditioning have opposite effects on chemotactic responses toward those chemicals.” (line 117-118, page 7)

4. *Lines 353-354: Figures 5B and 5C appear to be mislabeled.*

We checked the figure reference mentioned by the reviewer. This sentence is an explanation of Na+ chemotaxis phenotypes in the pkc-1 mutant, which are presented in Fig 5B. Therefore, we believe not to be mislabeled.

5. *Line 559: The use of rgef-1 is still strange. Please explain why you intended to do so.*

We added an explanation of why we used the rgef-1 promoter for deletion mutant isolation as follows. “We note that it has been found that *Cas9* expression by the *rgef-1* promoter, which is known to drive expression in the nervous system, could yield deletion mutants in the process of generating neuron-specific gene knockdown strains based on the somatic CRISPR/Cas9 system.” (line 590-593, page 39)

**--- Reviewer 3**

*The authors have adequately addressed my main concerns.*

*One minor concern remains. The legend of Figure 7 is insufficient to comprehend the model.*

1. *An increase in [Na] activates ASEL. Why isn’t it shown to promote attraction to [Na] in naive worms?*

In our previous paper (ref. 11, doi: 10.1523/JNEUROSCI.1774-16.2017.), we have shown that ASEL shows little or no response to Na^+^ in animals grown in NaCl-free conditions by calcium imaging experiments, consistent with no behavioral response to Na+ in naïve animals. We added this description in the caption of Figure 7 and deleted the dashed line on the left side in the figure to avoid confusion (line 465-467, page 32).

2. *What is the meaning of the orange squiggles? Why do they sometimes appear on the left, sometimes on right of the rectangles with which they are associated?*

The orange squiggles were placed near the sensory responses, which were experimentally confirmed in this paper or our previous paper (ref. 11). In the revised manuscript, we deleted the squiggles, because they could cause confusion. (Figure 7)

3. *What is significance of arrow line color and style (dashed versus solid)?*

Black lines indicate experimentally confirmed in this paper or the previous paper (ref. 11), and gray lines indicate experimentally not confirmed yet. We added this information to the Figure 7 caption.
