# Supplementary material for: Antagonistic regulation of salt and sugar chemotaxis plasticity by a single chemosensory neuron in Caenorhabditis elegans
Source: PLoS Genet. 2023 Sep 5;19(9):e1010637. doi: 10.1371/journal.pgen.1010637 (PMC10503759; doi:10.1371/journal.pgen.1010637)
Supplement: Peer review history

# Peer review: Decision Letter 0

**DOI:** 10.1371/journal.pgen.1010637.r001

**Authors:** Anne C. Hart, Gregory P. Copenhaver

12 Mar 2023

Dear Dr Tomioka,

Thank you very much for submitting your Research Article entitled 'Antagonistic regulation of salt and sugar chemotaxis plasticity by a single chemosensory neuron in Caenorhabditis elegans' to PLOS Genetics.

The manuscript was fully evaluated at the editorial level and by independent peer reviewers. The reviewers appreciated the attention to an important problem, but they raised some substantial concerns about the current manuscript. Based on the reviews, we will not be able to accept this version of the manuscript, but we would be willing to review a much-revised version. We cannot, of course, promise publication at that time. 

Should you decide to revise the manuscript for further consideration here, your revisions should address the specific points made by each reviewer. We will also require a detailed list of your responses to the review comments and a description of the changes you have made in the manuscript. Please note that based on PLOS Genetics data availability policy (below), we require that all numerical data and statistical analysis underlying results presented in a manuscript be made permanently accessible for readers, either in a comprehensive supplementary data worksheet accompanying your revised manuscript or in a permanent and open-access location on the internet. 

If you decide to revise the manuscript for further consideration at PLOS Genetics, please aim to resubmit within the next 60 days, unless it will take extra time to address the concerns of the reviewers, in which case we would appreciate an expected resubmission date by email to plosgenetics@plos.org.

If present, accompanying reviewer attachments are included with this email; please notify the journal office if any appear to be missing. They will also be available for download from the link below. You can use this link to log into the system when you are ready to submit a revised version, having first consulted our Submission Checklist.

To enhance the reproducibility of your results, we recommend that you deposit your laboratory protocols in protocols.io, where a protocol can be assigned its own identifier (DOI) such that it can be cited independently in the future. Additionally, PLOS ONE offers an option to publish peer-reviewed clinical study protocols. Read more information on sharing protocols at https://plos.org/protocols?utm_medium=editorial-email&utm_source=authorletters&utm_campaign=protocols

Please be aware that our data availability policy requires that all numerical data underlying graphs or summary statistics are included with the submission, and you will need to provide this upon resubmission if not already present. In addition, we do not permit the inclusion of phrases such as "data not shown" or "unpublished results" in manuscripts. All points should be backed up by data provided with the submission.

While revising your submission, please upload your figure files to the Preflight Analysis and Conversion Engine (PACE) digital diagnostic tool.  PACE helps ensure that figures meet PLOS requirements. To use PACE, you must first register as a user. Then, login and navigate to the UPLOAD tab, where you will find detailed instructions on how to use the tool. If you encounter any issues or have any questions when using PACE, please email us at figures@plos.org.

PLOS has incorporated Similarity Check, powered by iThenticate, into its journal-wide submission system in order to screen submitted content for originality before publication. Each PLOS journal undertakes screening on a proportion of submitted articles. You will be contacted if needed following the screening process.

To resubmit, use the link below and 'Revise Submission' in the 'Submissions Needing Revision' folder.

We are sorry that we cannot be more positive about your manuscript at this stage. Please do not hesitate to contact us if you have any concerns or questions.

Yours sincerely,

Anne C. Hart

Academic Editor

PLOS Genetics

Gregory P. Copenhaver

Editor-in-Chief

PLOS Genetics

Reviewer's Responses to Questions

**Comments to the Authors:**

**Please note here if the review is uploaded as an attachment.**

Reviewer #1: Summary:

In this manuscript, Tomioka et al describe how C. elegans coordinates chemotaxis in response to salt and sugar. Using chemotaxis assays, calcium imaging and neuron-specific ablation, the authors show that the ASEL sensory neuron is central to these responses. They found that when animals are exposed to monosaccharides in the presence of food, they are attracted to the sugar but avoid it without such preconditioning. They further found that glucose conditioning reduces salt chemotaxis and salt conditioning reduces glucose chemotaxis. The authors subsequently identified molecules, by a candidate gene approach as well as a forward genetic screen, that act in ASEL to control these chemotaxis behaviors. Finally, I found that the data reported in the paper justify the conclusions drawn.

In general, the manuscript s clearly written (with a few exceptions detailed below) with high quality and scientific rigor. The abstract and author summary are concise, accurate, and accessible to field specialists.

Major Comments:

Does conditioning with any monosaccharide cause attraction? e.g. Fructose conditioning and glucose attraction or vice versa?

Section starting Line 146 - The rationale for examining the ASEs is not well justified. Why not examine any other sensory neuron? Did the authors test any other neuron(s)?

The authors tested the role of synaptic neurotransmission in glucose responses but not neuropeptides using an unc-31 mutant. This needs to be addressed.

More context in the discussion regarding the importance of such mechanisms in the worms' natural environment

Minor Comments:

Line 84 change ‘whose sensing’ to ‘ the sensing of which’

Line 111 change ‘on agar’ to ‘on an agar’

Line 149-151 this senstence needs to eb re-worked as it is very confusing ‘Left- and right-sided ASE neurons, ASEL and ASER, respectively, receive different ions and

respond to increase and decrease, respectively, in concentrations of ions to promote attraction to those ions [19, 22].’

Line 154 ‘rapidly ceased’ reads weird. Would reduced be better?

Line 183 change ‘the wild type’ to ‘wild type’

Line 201 ‘By contrast, it decreased in worms after glucose conditioning’ - what do the authors refer to ‘it’ - define what ‘it’ is.

Line 267 - change ‘the dyf-11 mutant, in which sensory cilium is rescued only in ASEL.’ to ‘the dyf-11 mutant, in which dyf-11 expression, and likely cilia function, is rescued only in ASEL’

Line 276 - define ‘they’

Line 370 remove the word ‘the’ before monosaccharides

The use of the word authentic promoter is not usual. I presume the authors mean the dyf-11 promoter here. I suggest just calling in the dyf-11 promoter to avoid confusion.

Figure 1A - the number of worms used per assay is not clear

Methods -

Two plasmid DNAs, including Cas9 cDNA under the rgef-1 promoter or ensa-1 sgRNA under the U6 promoter - this doesn’t sound correct. The rgef-1 promoter drives expression in neurons and not the germline.

Reviewer #2: Summary:

The authors discovered that C. elegans exhibits chemotaxis to monosaccharides and that its preference can be modified through conditioning. This result was unexpected and interesting, and the experiments were well-designed. However, there are a few questionable interpretations of the results. Moreover, the writing of the paper, particularly the Introduction, is poor. Overall, I think this work could be published when it is rewritten properly. Still, I would like to say that I like manuscripts that are carefully prepared even if it is a first submission.

Major comments:

Major comments on experiments

1) The results of Fig. 3A and 3B of ATR+ do not seem to match. I agree that light stimulation significantly increases the forward probability in the naive condition. However, the conditioned result does not look so.

2) I do not agree with the interpretation of the results in Fig. 5. The authors wrote that "PKC-1 function is required for avoidance of Na+ after glucose conditioning." However, (1) a simple interpretation of Fig. 5C and D suggests that the role of pkc-1 is to suppress Na+-taxis in the naive state, and that salt conditioning suppresses the function of PKC-1, allowing Na+-taxis to occur. Furthermore, (2) based on Fig. 5A, B, it can be inferred that the function of pkc-1 is not involved in the response to glucose in the naive state, but rather, it induces attraction to glucose after conditioning.　　

3) Fig. 6C: If we simply predict the double mutant phenotype, all indices may be around 0.5-0.7, but that was not the case. Of course, experiments do not always go as expected. Still, I would like an explanation of the possible interpretations.

Minor comments for experiments

1) line 86 should be read as: "Although naive worms avoid these monosaccharides, they are attracted..."

2) line 118: Does "after feeding conditioning without glucose" mean naive?

3) line 167: Original paper(s) for dyf-11 cloning should be cited as well.

4) line 212: "after increasing and decreasing glucose concentration" was not described in the Figure.

5) line 339: How about changing Fig. 6A and B?

6) line 360: Which promoters were used in Fig. 6D?

7) line 431: Why was the food bacteria changed? Wouldn't this result in a loss of consistency between behavior and calcium imaging?

8) line 444: Add "in total".

9) line 447: I am unfamiliar with CRISPR/Cas system, but I couldn't understand why cas9 cDNA needs to be expressed under rgef-1 promoter for neurons.

10) line 508: Please describe the company name and cat. no. for the ring LED.

11) Fig. 2A: Adding the glucose concentration info will be helpful.

12) Fig. 2C: What does "authentic" mean?

Writing:

(1) The first paragraph of the Introduction seems to be about the general sensory reception of animals, but all the references cited are for C. elegans. This is strange. The references should either include more sources other than C. elegans, or make it clear that the focus is solely on C. elegans from the beginning.

(2) The second paragraph of the Introduction describes C. elegans' sensory reception, particularly regarding NaCl. However, only papers from the authors' group were cited, despite excellent results on this topic from many other research labs.

(3) The last paragraph of the Introduction is problematic. I recommend authors to read "Science Research Writing For Non-Native Speakers Of English" by Hilary Glasman-Deal.

(4) The way the figure legends are placed is also inadequate. Generally, the main figure legends should come after the main text. If they are inserted in the main text, they should be placed together with the figures. Additionally, the Sup. Fig. legend should be on the same page as the Sup. Fig.

Reviewer #3: Tomioka et al. describe an interesting new aspect of classical conditioning in C. elegans with food as the US. Using glucose as the CS, which is normally aversive, the authors find that conditioning reverses the worm's baseline avoidance of this compound. Interestingly, glucose-food causes NaCl, which is normally attractive, to become aversive. This suggests an adaptation for heightened sensitivity to food-predictive cues based on experience. The authors also provide some evidence for downstream molecular signaling pathways that may be involved.

Main concerns are:

Statistical methodology may be incorrect in a number of instances.

The Discussion lacks a clear summary of the working model this paper substantiates.

The paper is hard to read as it is quite terse, with intervening steps of experimental logic and interpretation left for the reader to discover.

MAJOR

Figs. 2C, 4A, 4B, 4C, 5A, 5B, 6D, etc. The authors frequently use statistical comparisons between treatment groups to demonstrate the presence or absence of attraction or repulsion. These seem incorrect. The appropriate test would be difference from zero (see Fig. 1A). This is a non-trivial matter as some of the author's claims may turn out to be unsupported by the data when the appropriate statistics are used. For example, the claim of Glc attraction in unconditioned egl-30 may not hold up (Fig. 5A). The same concern applies to the claim of Glc attraction in unconditioned animals expressing PKC-1(gf) in ASEL (Fig. 5B). The authors should check all figures for other instances of this error.

Fig. 3A conditioned. The claim of reduced forward probability in the conditioned group upon ChR2 activation of ASEL looks like it might be an artifact of the analysis. The authors computed the difference between mean P-forward in the entire prestimulus baseline period and mean P-forward during the stimulus. This difference is amplified by the fact that the prestimulus baseline is elevated for some reason. If, instead, the authors took as the prestimulus baseline the 5 sec period before the stimulus, the effect would likely disappear. Fig. 3A Naive. Could the authors please discuss the apparent rebound effect after the stimulus. Maybe there's a rebound effect in the Naive group as well. Perhaps these issues could be clarified by showing the full 40 sec of pre- and poststimulus data.

Discussion. This section might be improved by a summary figure showing how the effects of conditioning on ASEL interact with the PKC-1 and ENSA-1 pathways to regulate attraction and repulsion. See also comment below (regarding lines 305-310).

MINOR

56-67. The theme of sensory motor integration, mentioned in the introduction, is under developed in the main text.

Could the authors please explain why they find repulsion rather than attraction to glucose, as previously reported in reference [20]

189 and captions. "Authentic" promoter is confusing. Maybe "native" promoter would be more clear.

212. "Pirouette frequency increased and decreased after increasing and decreasing glucose concentration" is a bit confusing. Strictly speaking, Fig. 3C shows that pirouette frequency was above baseline (at dC/dt = 0) for dC/dt > 0 and below baseline for dC/dt < 0.

240. "dC/dt rank" is confusing, because nothing was ranked. Rather, there are two DOMAINS, dC/dt > 0 and dC/dt < 0, and the pirouette index is the difference in mean pirouette frequency in these two domains.

272. Should this read "(Fig. 4C, THIRD block)"?

273-4. Indicate which figure panel contains the data supporting this conclusion.

Fig. 4C. Please explain why the authentic and ASEL-selective promoters give different results in dyf-11 rescue.

277. Please explain how the results with 2-ASEL strain imply something about ASER function.

305-310. Can the authors please provide a conceptual model of how increased/decreased synaptic output from ASEL or ASER explains the changes in chemotaxis?

339. "Increased Na attraction" relative to what other treatment group?

351. The authors note that PKC-1 is localized to the presynaptic region in ASER. Where is PKC-1 expressed in ASEL?

352. The axon in Fig. 6E is not visible.

371. What might be the source of monosaccharides in the natural environment? Would bacteria be associated with them?

400. in _an_ opposite direction.

Fig. 1. Concentration of Na and sugars during conditioning is unclear.

Fig. 2A top. The authors might consider using an ANOVA with repeated measures to test for the effect of conditioning. This avoids the potential bias introduced by arbitrarily defining peak and non-peak regions in the traces.

Fig. 2C Add the label "Promoter" to the line below the x-axis containing "authentic," "ASEL," "ASER."

Fig. 3C-G. How was dC/dt computed?

Fig. 3H. Y-axis is unlabeled. It should be pirouette index?

Fig. 4C Add "Promoter" to x-axis label.

Fig. 5B, first block. These data appear to be the same as Fig. 2C. This should be stated; same for any other re-use of data.

Fig. 5B, key is cutoff.

Fig. 1A,B; 5C,D, control groups. There seems to be no chemotaxis to Na ions, which are normally attractive.

Fig. 6A,B. Might be good to reverse the order of these panels to match the text order.

Fig. 6C. Could the authors please explain in what respect the double mutant has a stronger phenotype than the single mutants? This is needed to support their claim of parallel/redundant pathways.

The authors make the case that worms can use sensation of monosaccharides as a cue signaling the presence of food. Can worms eat/digest environmental sugars?

**********

**Have all data underlying the figures and results presented in the manuscript been provided?**

Large-scale datasets should be made available via a public repository as described in the *PLOS Genetics* data availability policy, and numerical data that underlies graphs or summary statistics should be provided in spreadsheet form as supporting information.

Reviewer #1: Yes

Reviewer #2: **No: **In my understanding, the numerial data was not provided.

Reviewer #3: None

**********

PLOS authors have the option to publish the peer review history of their article (what does this mean?). If published, this will include your full peer review and any attached files.

If you choose “no”, your identity will remain anonymous but your review may still be made public.

**Do you want your identity to be public for this peer review?** For information about this choice, including consent withdrawal, please see our Privacy Policy.

Reviewer #1: No

Reviewer #2: No

Reviewer #3: No

# Author comment: Author response to Decision Letter 0

**DOI:** 10.1371/journal.pgen.1010637.r002

22 May 2023

Attachment Submitted filename: Response letter_Tomioka_et_al_final3.docx Click here for additional data file.

# Peer review: Decision Letter 1

**DOI:** 10.1371/journal.pgen.1010637.r003

**Authors:** Anne C. Hart, Gregory P. Copenhaver

4 Jul 2023

Dear Dr Tomioka,

Thank you very much for submitting your Research Article entitled 'Antagonistic regulation of salt and sugar chemotaxis plasticity by a single chemosensory neuron in Caenorhabditis elegans' to PLOS Genetics.

The manuscript was fully evaluated at the editorial level and by independent peer reviewers. The reviewers appreciated the revisions and the attention to an important topic, but they identified some concerns that we ask you address in a revised manuscript. It seems likely that only revision of the text and figures would be needed to address the remaining concerns;  as academic editor I believe that additional experiments are not required. 

We therefore ask you to modify the manuscript according to the review recommendations below. Your revisions should address the specific points made by each reviewer. 

In addition we ask that you:

1) Provide a detailed list of your responses to the review comments and a description of the changes you have made in the manuscript.

2) Upload a Striking Image with a corresponding caption to accompany your manuscript if one is available (either a new image or an existing one from within your manuscript). If this image is judged to be suitable, it may be featured on our website. Images should ideally be high resolution, eye-catching, single panel square images. For examples, please browse our archive. If your image is from someone other than yourself, please ensure that the artist has read and agreed to the terms and conditions of the Creative Commons Attribution License. Note: we cannot publish copyrighted images.

We hope to receive your revised manuscript within the next 30 days. If you anticipate any delay in its return, we would ask you to let us know the expected resubmission date by email to plosgenetics@plos.org.

If present, accompanying reviewer attachments should be included with this email; please notify the journal office if any appear to be missing. They will also be available for download from the link below. You can use this link to log into the system when you are ready to submit a revised version, having first consulted our Submission Checklist.

While revising your submission, please upload your figure files to the Preflight Analysis and Conversion Engine (PACE) digital diagnostic tool. PACE helps ensure that figures meet PLOS requirements. To use PACE, you must first register as a user. Then, login and navigate to the UPLOAD tab, where you will find detailed instructions on how to use the tool. If you encounter any issues or have any questions when using PACE, please email us at figures@plos.org.

Please be aware that our data availability policy requires that all numerical data underlying graphs or summary statistics are included with the submission, and you will need to provide this upon resubmission if not already present. In addition, we do not permit the inclusion of phrases such as "data not shown" or "unpublished results" in manuscripts. All points should be backed up by data provided with the submission.

To enhance the reproducibility of your results, we recommend that you deposit your laboratory protocols in protocols.io, where a protocol can be assigned its own identifier (DOI) such that it can be cited independently in the future. Additionally, PLOS ONE offers an option to publish peer-reviewed clinical study protocols. Read more information on sharing protocols at https://plos.org/protocols?utm_medium=editorial-email&utm_source=authorletters&utm_campaign=protocols

Please review your reference list to ensure that it is complete and correct. If you have cited papers that have been retracted, please include the rationale for doing so in the manuscript text, or remove these references and replace them with relevant current references. Any changes to the reference list should be mentioned in the rebuttal letter that accompanies your revised manuscript. If you need to cite a retracted article, indicate the article’s retracted status in the References list and also include a citation and full reference for the retraction notice.

PLOS has incorporated Similarity Check, powered by iThenticate, into its journal-wide submission system in order to screen submitted content for originality before publication. Each PLOS journal undertakes screening on a proportion of submitted articles. You will be contacted if needed following the screening process.

To resubmit, you will need to go to the link below and 'Revise Submission' in the 'Submissions Needing Revision' folder.

Please let us know if you have any questions while making these revisions.

Yours sincerely,

Anne C. Hart

Academic Editor

PLOS Genetics

Gregory P. Copenhaver

Editor-in-Chief

PLOS Genetics

 

**--- Reviewer 1**

The authors have satisfactorily answered my initial concerns except for the justification for examining the role of the ASEs. In response to my original question the authors state "In fact, we first conducted cell-specific rescue experiments using the sensory cilia-defective dyf-11 mutant to explore chemosensory neurons important for glucose chemotaxis." and "We also added the result of glucose chemotaxis using the dyf-11 mutant in which dyf-11 function (cilium function) is rescued in chemosensory neurons other than ASE by the odr-4 promoter"

However, it is not at all clear why the authors examined ASE function. Why not any other chemosensory neuron? As the authors performed cell-specific rescue experiments for other neurons the why not present these data? The jump from using the dyf-11 and odr-4 promoters and then looking at the ASEs is unclear. This needs to be explained better and/or the cell-specific data shown.

**--- Reviewer 2**

The authors have adequately addressed most of my previous comments. Unfortunately, however, I still have a few comments:

Relatively major:

1. Line 404: I do not agree that the effects of pkc-1 and ensa-1 are additive. The double seems statistically similar to pkc-1.

2. Line 407: I suppose that pkc-1 and ensa-1 play redundant roles, and that a lack of both causes the apparent phenotype.

3. Figure 7: According to Figure 5, pkc-1 and ensa-1 also seem to play roles in the naive condition, which should be properly shown in the drawing.

Minor:

1. Line 61: Please consider using "external" instead of "sensory".

2. The 2nd and 3rd paragraphs in the Introduction: I found it difficult to understand how these paragraphs are related to the results of this manuscript. Adding topic sentences might help clarify this connection.

3. Line 113: Could you explain why you consider the information is processed separately?

4. Lines 353-354: Figures 5B and 5C appear to be mislabeled.

5. Line 559: The use of rgef-1 is still strange. Please explain why you intended to do so.

**--- Reviewer 3**

The authors have adequately addressed my main concerns.

One minor concern remains. The legend of Figure 7 is insufficient to comprehend the model.

1. An increase in [Na] activates ASEL. Why isn’t it shown to promote attraction to [Na] in naive worms?

2. What is the meaning of the orange squiggles? Why do they sometimes appear on the left, sometimes on right of the rectangles with which they are associated?

3. What is significance of arrow line color and style (dashed versus solid)?

# Author comment: Author response to Decision Letter 1

**DOI:** 10.1371/journal.pgen.1010637.r004

3 Aug 2023

Attachment Submitted filename: Response letter-fin-2.docx Click here for additional data file.

# Peer review: Decision Letter 2

**DOI:** 10.1371/journal.pgen.1010637.r005

**Authors:** Anne C. Hart, Gregory P. Copenhaver

20 Aug 2023

Dear Dr Tomioka,

We are pleased to inform you that your manuscript entitled "Antagonistic regulation of salt and sugar chemotaxis plasticity by a single chemosensory neuron in Caenorhabditis elegans" has been editorially accepted for publication in PLOS Genetics. Congratulations!

Before your submission can be formally accepted and sent to production you will need to complete our formatting changes, which you will receive in a follow up email. Please be aware that it may take several days for you to receive this email; during this time no action is required by you. Please note: the accept date on your published article will reflect the date of this provisional acceptance, but your manuscript will not be scheduled for publication until the required changes have been made.

Once your paper is formally accepted, an uncorrected proof of your manuscript will be published online ahead of the final version, unless you’ve already opted out via the online submission form. If, for any reason, you do not want an earlier version of your manuscript published online or are unsure if you have already indicated as such, please let the journal staff know immediately at plosgenetics@plos.org.

In the meantime, please log into Editorial Manager at https://www.editorialmanager.com/pgenetics/, click the "Update My Information" link at the top of the page, and update your user information to ensure an efficient production and billing process. Note that PLOS requires an ORCID iD for all corresponding authors. Therefore, please ensure that you have an ORCID iD and that it is validated in Editorial Manager. To do this, go to ‘Update my Information’ (in the upper left-hand corner of the main menu), and click on the Fetch/Validate link next to the ORCID field.  This will take you to the ORCID site and allow you to create a new iD or authenticate a pre-existing iD in Editorial Manager.

If you have a press-related query, or would like to know about making your underlying data available (as you will be aware, this is required for publication), please see the end of this email. If your institution or institutions have a press office, please notify them about your upcoming article at this point, to enable them to help maximise its impact. Inform journal staff as soon as possible if you are preparing a press release for your article and need a publication date.

Thank you again for supporting open-access publishing; we are looking forward to publishing your work in PLOS Genetics!

Yours sincerely,

Anne C. Hart

Academic Editor

PLOS Genetics

Gregory P. Copenhaver

Editor-in-Chief

PLOS Genetics

www.plosgenetics.org

Twitter: @PLOSGenetics

----------------------------------------------------

Comments from the reviewers (if applicable):

Reviewer's Responses to Questions

**Comments to the Authors:**

**Please note here if the review is uploaded as an attachment.**

Reviewer #1: My original concerns have been satisfactorily addressed.

Reviewer #2: The authors have adequately addressed the previous reviewer comments. I think the manuscript is acceptable for publication.

Reviewer #3: Approved

**********

**Have all data underlying the figures and results presented in the manuscript been provided?**

Large-scale datasets should be made available via a public repository as described in the *PLOS Genetics* data availability policy, and numerical data that underlies graphs or summary statistics should be provided in spreadsheet form as supporting information.

Reviewer #1: Yes

Reviewer #2: Yes

Reviewer #3: None

**********

PLOS authors have the option to publish the peer review history of their article (what does this mean?). If published, this will include your full peer review and any attached files.

If you choose “no”, your identity will remain anonymous but your review may still be made public.

**Do you want your identity to be public for this peer review?** For information about this choice, including consent withdrawal, please see our Privacy Policy.

Reviewer #1: No

Reviewer #2: No

Reviewer #3: No

----------------------------------------------------

**Data Deposition**

If you have submitted a Research Article or Front Matter that has associated data that are not suitable for deposition in a subject-specific public repository (such as GenBank or ArrayExpress), one way to make that data available is to deposit it in the Dryad Digital Repository. As you may recall, we ask all authors to agree to make data available; this is one way to achieve that. A full list of recommended repositories can be found on our website.

The following link will take you to the Dryad record for your article, so you won't have to re‐enter its bibliographic information, and can upload your files directly: 

http://datadryad.org/submit?journalID=pgenetics&manu=PGENETICS-D-23-00092R2

More information about depositing data in Dryad is available at http://www.datadryad.org/depositing. If you experience any difficulties in submitting your data, please contact help@datadryad.org for support.

Additionally, please be aware that our data availability policy requires that all numerical data underlying display items are included with the submission, and you will need to provide this before we can formally accept your manuscript, if not already present.

----------------------------------------------------

**Press Queries**

If you or your institution will be preparing press materials for this manuscript, or if you need to know your paper's publication date for media purposes, please inform the journal staff as soon as possible so that your submission can be scheduled accordingly. Your manuscript will remain under a strict press embargo until the publication date and time. This means an early version of your manuscript will not be published ahead of your final version. PLOS Genetics may also choose to issue a press release for your article. If there's anything the journal should know or you'd like more information, please get in touch via plosgenetics@plos.org.

# Editor report: Acceptance letter

**DOI:** 10.1371/journal.pgen.1010637.r006

**Authors:** Anne C. Hart, Gregory P. Copenhaver

31 Aug 2023

PGENETICS-D-23-00092R2 

Antagonistic regulation of salt and sugar chemotaxis plasticity by a single chemosensory neuron in Caenorhabditis elegans 

Dear Dr Tomioka, 

We are pleased to inform you that your manuscript entitled "Antagonistic regulation of salt and sugar chemotaxis plasticity by a single chemosensory neuron in Caenorhabditis elegans" has been formally accepted for publication in PLOS Genetics! Your manuscript is now with our production department and you will be notified of the publication date in due course.

The corresponding author will soon be receiving a typeset proof for review, to ensure errors have not been introduced during production. Please review the PDF proof of your manuscript carefully, as this is the last chance to correct any errors. Please note that major changes, or those which affect the scientific understanding of the work, will likely cause delays to the publication date of your manuscript. 

Soon after your final files are uploaded, unless you have opted out or your manuscript is a front-matter piece, the early version of your manuscript will be published online. The date of the early version will be your article's publication date. The final article will be published to the same URL, and all versions of the paper will be accessible to readers.

Thank you again for supporting PLOS Genetics and open-access publishing. We are looking forward to publishing your work! 

With kind regards,

Anita Estes

PLOS Genetics

On behalf of:

The PLOS Genetics Team

Carlyle House, Carlyle Road, Cambridge CB4 3DN | United Kingdom

plosgenetics@plos.org | +44 (0) 1223-442823

plosgenetics.org | Twitter: @PLOSGenetics
